# 539 Contact Burns Associated with Concomitant use of Methamphetamines and Heat Stroke, Resulting in Neurological Sequela

**DOI:** 10.1093/jbcr/irad045.136

**Published:** 2023-05-15

**Authors:** Colleen Glennon, Karen Richey, Kevin Foster

**Affiliations:** Arizona Burn Center Valleywise Health, Phoenix, Arizona; Arizona Burn Center Valleywise Health, Phoenix, Arizona; AZ Burn Center at Valleywise Health, Phoenix, Arizona; Arizona Burn Center Valleywise Health, Phoenix, Arizona; Arizona Burn Center Valleywise Health, Phoenix, Arizona; AZ Burn Center at Valleywise Health, Phoenix, Arizona; Arizona Burn Center Valleywise Health, Phoenix, Arizona; Arizona Burn Center Valleywise Health, Phoenix, Arizona; AZ Burn Center at Valleywise Health, Phoenix, Arizona

## Abstract

**Introduction:**

On a summer day in the desert southwest, ground surfaces can soar to 180 degrees Fahrenheit, posing significant risk to individuals who sustain contact with such surfaces, particularly if unable to get up. These individuals are also at risk for heat stroke. This summer, an increased number of patients admitted with contact burns were positive for methamphetamine and concomitant heat stroke. The purpose of this case series is to describe the hospital course and outcomes of this subset of patients.

**Methods:**

A retrospective chart review of patients admitted between June and September 2022 was completed. Criteria for inclusion included contact burn, initial core body temperature > 38.8°C, and a methamphetamine positive drug screen. Outcome measures included length of stay, discharge disposition, number of operations, admission Glasgow Coma Score, neurology work-up and sequela.

**Results:**

A total of 23 patients were admitted who suffered contact burns and tested positive for methamphetamine. Ten of those patients had core body temperatures greater than 38.8°C upon initial evaluation. The mean age was 36.6 years (24-62), with an average temperature of 42.3°C (40.5-43.1°. All 10 were found down in the community with altered mental status, the average Glasgow Coma Scale was 5.9 (3- 12). CT scans were done on 9, all were negative for acute intracranial abnormalities. Neurology was consulted for 3 patients with 2 having EEGs performed, both displayed slowing and attenuation secondary to hyperthermia. Ten of these patients were concurrently positive for fentanyl. The mean total burn surface area burned was 9.6% (4-17.5%), The average number of operations was 4.1/patient (1-11). Of the 8 patients discharged the average LOS was 30 days (10-61), 2 patients remain hospitalized in the ICU with a current LOS of 61-71 days. Of the 8 patients discharged, all required post-acute care with only 1 meeting rehabilitation criteria.

**Conclusions:**

Elevated temperatures in the Southwestern United States have been known to cause an increase in surface-related contact burns during the summer months. Historically, the at-risk patient population has been elderly individuals prone to falls with limited physical ability to get up. This summer highlighted a new phenomenon of younger individuals suffering both contact burns and heat stroke secondary to the combination of methamphetamine use and elevated summer temperatures. This patient population suffered significant morbidity, and none were able to be discharged home. This case series highlights the dangers posed by ambiently heated surfaces during the summer months, particularly in combination with illicit drug use.

**Applicability of Research to Practice:**

This case series, demonstrates the increased risk of sequela associated with burn patients who have comorbid substance abuse disorder.

